# Plant hairy root cultures as plasmodium modulators of the slime mold emergent computing substrate *Physarum polycephalum*

**DOI:** 10.3389/fmicb.2015.00720

**Published:** 2015-07-16

**Authors:** Vincent Ricigliano, Javed Chitaman, Jingjing Tong, Andrew Adamatzky, Dianella G. Howarth

**Affiliations:** ^1^Department of Biological Sciences, St. John's UniversityNew York, NY, USA; ^2^Unconventional Computing Centre, University of the West of EnglandBristol, UK

**Keywords:** *Valeriana officinalis*, Physarum polycephalum, hairy root cultures, chemotaxis, *Agrobacterium rhizogenes*, plant transformation, electrical activity

## Abstract

Roots of the medicinal plant *Valeriana officinalis* are well-studied for their various biological activities. We applied genetically transformed *V. officinalis* root biomass to exert control of *Physarum polycephalum*, an amoeba-based emergent computing substrate. The plasmodial stage of the *P. polycephalum* life cycle constitutes a single, multinucleate cell visible by unaided eye. The plasmodium modifies its network of oscillating protoplasm in response to spatial configurations of attractants and repellents, a behavior that is interpreted as biological computation. To program the computing behavior of *P. polycephalum*, a diverse and sustainable library of plasmodium modulators is required. Hairy roots produced by genetic transformation with *Agrobacterium rhizogenes* are a metabolically stable source of bioactive compounds. Adventitious roots were induced on *in vitro V. officinalis* plants following infection with *A. rhizogenes*. A single hairy root clone was selected for massive propagation and the biomass was characterized in *P. polycephalum* chemotaxis, maze-solving, and electrical activity assays. The *Agrobacterium*-derived roots of *V. officinalis* elicited a positive chemotactic response and augmented maze-solving behavior. In a simple plasmodium circuit, introduction of hairy root biomass stimulated the oscillation patterns of slime mold's surface electrical activity. We propose that manipulation of *P. polycephalum* with the plant root culture platform can be applied to the development of slime mold microfluidic devices as well as future models for engineering the plant rhizosphere.

## Introduction

The dominant life-form of the slime mold *Physarum polycephalum* (Physaridae) is a large, polynuclear plasmodium, which transports nutrients and chemical signals through a continuous arrangement of oscillating protoplasm. This giant amoeba forages by deploying a mass of pseudopods to discover multiple nutrient sources and connects them via a network of protoplasmic tubes (Keller et al., 1995). The foraging behavior of the *P. polycephalum* plasmodium can be interpreted as a computation; data are represented by spatial configurations of attractants and repellents, and results are represented by the structure of the protoplasmic network. *P. polycephalum* can perform complex computational tasks with natural parallelism, such as maze solving (Nakagaki et al., 2000a), calculation of optimal graphs (Adamatzky, 2009a), sub-division of spatial configurations of data points, construction of arithmetic circuits and logic gates (Tsuda et al., 2004), and robot control (Tsuda et al., 2009). The protoplasmic network reorganizes in response to extracellular stimuli and excitation waves propagating and interacting within the plasmodium (Nakagaki et al., 2000b). A plasmodium can be considered a reaction-diffusion medium, or an excitable medium (Achenbach and Weisenseel, 1981; Adamatzky, 2009b), encapsulated in a biological membrane, and has become a popular substrate for the design of emergent sensing and computing devices. *P. polycephalum*-based devices integrating circuits of living plasmodium with conventional electronic components have employed a variety of data and control inputs via chemical, optical, and mechanical means (Adamatzky, 2013b).

*P. polycephalum*'s auto-oscillatory mode of locomotion is a model for studying non-muscular motility, and plasmodium chemotactic behavior has been well-documented (Carlile, 1970; Durham and Ridgway, 1976; Knowles and Carlile, 1978). Numerous studies indicate that protoplasmic movement is controlled by an oscillatory system known as shuttle streaming with an inherent frequency of from 1 to 5 min; stimulation by both positive and negative chemotactic stimuli increase and decrease this frequency respectively (Kishimoto, 1958; Kamiya, 1981). Most chemotaxis studies of *P. polycephalum* have focused on simple carbohydrates with glucose, galactose, and mannose being the strongest chemoattractants (Carlile, 1970; Knowles and Carlile, 1978). Laboratory cultures are most commonly fed oat flakes, which consist of approximately 70% carbohydrate. Non-nutrient substances also exhibit chemotactic properties including cyclic 3′,5′-AMP phosphodiesterase enzyme inhibitors (Kincaid and Mansour, 1979). Recently, it was found that plasmodium exhibit a marked chemotactic response to volatile organic compounds including the plant isoprenoids farnesene, β-myrcene, and α-pinene (de Lacy Costello and Adamatzky, 2013). These findings suggest that volatile plant compounds might augment the plasmodium through unmapped mechanisms of action.

Establishing a diverse and reliable library of chemomodulators is fundamental for the development of *P. polycephalum* as a computing substrate. Our previous efforts to identify chemotactic agents of plant origin investigated the slime mold's binary choice between biologically active species including *Valeriana officinalis, Humulus lupulus, Passiflora incarnate, Lactuca virosa, Gentiana lutea*, and *Verbena officinalis*. A hierarchy of chemoattractive force was calculated, indicating that *P. polycephalum* had the strongest preference for powdered roots of *Valeriana officinalis* (*V. officinalis*) (Adamatzky, 2011). Herbal preparations of *V. officinalis* roots are widely recognized for their diverse pharmacological properties whereas ecologically relevant biological activity is comparatively unexplored (Patoèka and Jakl, 2010). Plants in the genus *Valeriana* exhibit root-specific accumulation of volatile oils and their isoprenoid derivatives. Considering this differential spatial accumulation of volatile oils, it is likely that these compounds are involved in rhizospheric interactions such as signaling to pathogens or symbionts. Plant roots impact the behavior and composition of microbial communities through exudation of organic compounds (Huang et al., 2014). In turn, plant-associated microorganisms influence plant development (Desbrosses and Stougaard, 2011) and specialized metabolism (Zhi-lin et al., 2007). It is possible that the slime mold response to *V. officinalis* root is due to a presently uncharacterized ecological association, or a crosstalk between convergent signaling pathways. Nevertheless, *V. officinalis* root tissues are a promising source of modulatory stimuli for exerting control of plasmodium physiology and behavior.

Plants synthesize a plethora of compounds that are not vital for basic cellular functions yet instrumental for mediating particular ecological interactions. These specialized metabolites typically occur in highly variable concentrations *in planta* due to variation in genetic and environmental factors (Dudareva et al., 2013). In order to control for *in planta* variation, plants would need to be grown in a controlled environment with equivalent genetic backgrounds. Fast growth rates and equivalent biosynthetic capacity to wild type plant roots make hairy root cultures a promising platform for stable metabolite production by plant tissue. Infection with the phytopathogen *Agrobacterium rhizogenes* and stable integration of root-inducing plasmid DNA into the host plant genome induces adventitious roots commonly referred to as hairy roots (Chandra and Chandra, 2011). Neoplastic root growth occurs via modification of plant hormone metabolism through the introduction of non-native enzymes (Nilsson and Olsson, 1997). Cultures of transformed roots possess genetic and biochemical stability (Guillon et al., 2006), enabling massive propagation of uniform biomass (Srivastava and Srivastava, 2007).

In this study we assayed the modulatory potential of *V. officinalis* hairy root cultures in *P. polycephalum*, a slime mold which exhibits many novel and attractive properties in the areas of unconventional computation and non-silicon hardware. This work aimed to advance the field of *P. polycephalum* synthetic biology by applying for the first time a bioactive plant *in vitro* culture system that is amenable to experimental manipulation.

## Materials and methods

### Culturing *P. polycephalum*

*P. polycephalum* plasmodia were maintained on 2% non-nutrient agar and incubated in darkness at 28 ± 2°C. The initial culture of wild type *P. polycephalum* plasmodium was obtained from Carolina Biological Supply (Burlington, North Carolina USA). Laboratory cultures were fed 200–400 mg of oat flakes daily and were subcultured to fresh media every 5 days. This culture method provided large quantities of individual oat flakes colonized with clonal *P. polycephalum* and facilitated inoculation of plasmodia with a short-term nutrient source into experimental assays.

### Establishment and culture of *V. officinalis* hairy roots

*V. officinalis* seedlings were aseptically grown at 25 ± 2°C with a 16 h light/day photoperiod on MS basal medium (Murashige and Skoog, 1962) containing 3% sucrose and solidified with 1% agar. *A. rhizogenes* (ATCC 15834) harboring root-inducing plasmid pRi15834 was used for the transformation experiments. Leaf segments of 1-month-old *V. officinalis* plants were submerged in *A. rhizogenes* suspension, blotted dry, and co-cultivated on hormone-free MS medium for 25 ± 2°C in the dark. After 3 days of co-cultivation, the explants were transferred to MS medium containing 200 mg/L timentin to kill the excess *Agrobacterium*. In 2–3 weeks, hairy roots emerged from callus on cut surfaces of the explants. Single root segments were removed from different explants to establish independent transformation events. Hairy root lines were maintained on hormone- and antibiotic-free semi-solid MS medium with subcultures performed every 3 weeks. Hairy root clone VoHR5 was selected based on vigorous growth and stable incorporation of *A. rhizogenes* root-inducing plasmid DNA into the plant genome. VoHR5 genomic DNA was used as template for PCR to detect the *rolB* gene from the *A. rhizogenes* pRi15834 T-DNA using the primers 5′- GCT CTT GCA GTG CTA GAT TT −3′ and 5′- GAA GGT GCA AGC TAC CTC TC −3′.

Clonal VoHR5 biomass was generated by propagation of the hairy root line in shake-flask culture array. Approximately 500 mg of VoHR5 root segments were inoculated into 30 ml of MS basal medium containing 3% sucrose in 150 ml shaker flasks. Cultures were maintained in darkness at 25 ± 2°C on orbital shakers at 120 rpm. The VoHR5 shake-flask cultures were harvested after 28 days and the material was extensively rinsed with sterile water, lyophilized, and stored at −80°C for no longer than 1 month until it was used in *P. polycephalum* bioassays.

### *P. polycephalum* chemotaxis and maze-solving assays

*V. officinalis* hairy root biomass was tested in the binary choice chemotactic assay we had previously used to establish *P. polycephalums*'s hierarchical preference for various biologically active plants (Adamatzky, 2011). Experiments were carried out in 100 × 15 mm petri dishes containing 2% non-nutrient agar. Two sample locations of 10 mm in diameter were bored into the media at the furthest points from the center on a straight line. A single oat flake colonized by *P. polycephalum* was placed at the center of the assay dish, on a straight line connecting the two sample locations and at the same distance from each sample. Uncolonized oat flake or 30 mg of powdered *V. officinalis* root (wild type or VoHR5 hairy root) biomass were added to the left and right sample locations in a randomized fashion. Assay plates were sealed with parafilm and incubated at 28 ± 2°C in darkness. At least twenty replicates were performed for each of the binary choice experiments and chemotactic behavior was recorded after 24 h. The criteria for behavioral assessment in each plate were according to predefined outcomes: if the plasmodium propagated horizontally from the site of inoculation toward a certain sample, a positive chemotactic event was recorded. If the plasmodium propagated toward both samples simultaneously, an equal preference event was recorded.

Maze-solving behavioral assays were carried out as previously described (Adamatzky, 2012): 70 × 3 mm plastic mazes containing 2% agar were used as the experimental substrate. An uncolonized oat flake or 30 mg of powdered VoHR5 hairy root biomass was placed in the center chamber of the maze with empty central chamber used as a control. An oat flake colonized with *P. polycephalum* was inoculated into the most peripheral channel of the maze. Mazes were sealed with parafilm to establish and maintain volatile gradients in the air paths of the maze and incubated at 28 ± 2°C in darkness. Twenty replicates of mazes containing either oat flake or hairy root in the central chamber were sampled at 24-, 36-, and 48- h periods after inoculation of the plasmodium into the assay.

### Measurement of *P. polycephalum* electrical activity

To measure the electrophysiological response to hairy root biomass, electrical activity of *P. polycephalum* plasmodium was recorded with ADC-24 High Resolution Data Logger (Pico Technology, UK). The data logger ADC-24 employs differential inputs, galvanic isolation and software-selectable sample rates, which contribute to noise-free resolution; its 24-bit A/D converter maintains a gain error of 0.1%, noise rejection of 120 dB typical at 50/60 Hz. Its input impedance is 2~ MOhm for differential inputs, and offset error is 36 mV in 1250 ~mV range use.

Oscillations of surface electrical potential were measured across a single protoplasmic tube spanning two electrodes. Each electrode was made of conductive aluminum foil, 0.07 mm thick, 8.0 mm wide, and 50 mm long. Two, 2 ml blobs of 3% agar were placed on electrodes mounted to the bottom of a 100 × 15 mm petri dish. The distance between proximal sites of electrodes was 10 mm. *P. polycephalum* was inoculated onto one agar blob and the assay plate was incubated at 28 ± 2°C for 24 h. During the incubation period, plasmodium colonized the first blob then propagated toward and colonized the second agar blob. Assay plates in which the electrical circuit was completed by a single protoplasmic tube spanning both electrodes were used for electrical activity measurements. Incomplete circuits, and assay plates where more than one tube connected the agar blobs were discarded. The electrode containing the initial colonized oat flake was connected to the ADC-24 analog ground. The electrode containing the newly colonized flake and agar blob was connected to an ADC-24 analog recording channel. Input channels were set to ± 39 mV ground referenced recording and sampling frequency of 2 Hz; simultaneous channels were recorded as required. This experimental design has proved reliable in studies of electrical activity of *P. polycephalum* (Whiting et al., 2014).

Testing of *V. officinalis* hairy root biomass was organized as follows: for each assay plate, electrical oscillatory activity of the plasmodium was recorded for 10 min to establish baseline levels. Then 30 mg of powdered VoHR5 hairy root biomass was loaded into a plastic cap and placed 20 mm from the electrode connected to the ADC-24 analog recording channel and oscillatory activity was recorded for 10 min. Dominating frequencies were calculated using fast fourier transform (SigmaView). At least thirty replicates were performed in which the electrical oscillation activity of plasmodium was recorded before and after the introduction of VoHR5 hairy root biomass.

### Statistical analysis

All probabilities were calculated using One-Way ANOVA with a Tukey's post-test with a 95% confidence interval (Graphpad Prism 3.03).

## Results and discussion

### *P. polycephalum* growth and behavior

**Figure 1:**
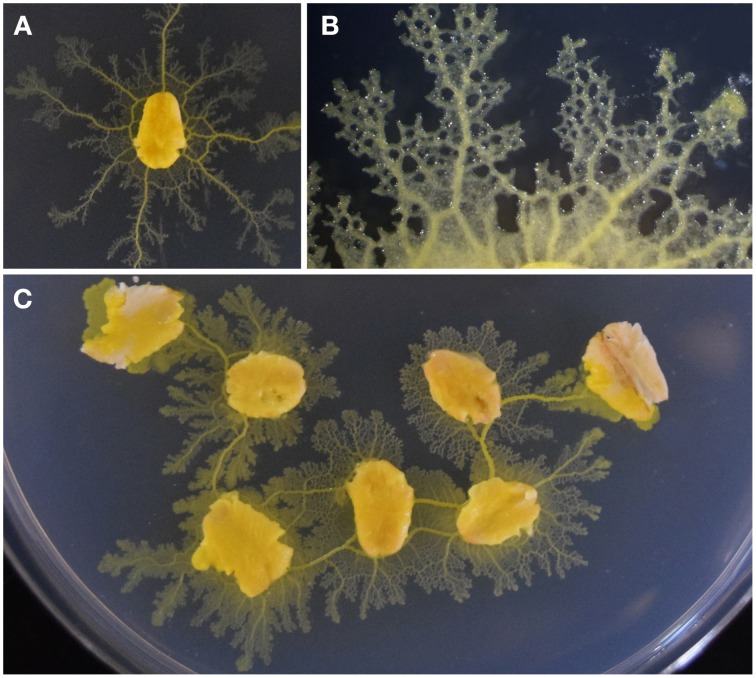
***P. polycephalum*** **growth and networking behavior as viewed from above**. **(A)** The organism extends concentrically from a colonized oat flake onto the agar substrate. **(B)** A growing tip of plasmodium and its network of protoplasmic tubes. **(C)** Foraging behavior of plasmodium showing the connection of multiple food sources. Thick tubes join the nodes of nutrients as the protoplasmic network develops.

The plasmodial stage of *P. polycephalum* resembles an elaborate network of tubular modules. When assimilating a new food source, morphological adaptations maintain the optimal cell volume required for the connecting protoplasmic tubes (Figure 1). If separated, a section of the protoplasmic network will restore functionality to its surrounding membrane, resuming contractile, and locomotive activities as a distinct cell. Therefore, fragments of clonal plasmodium are applicable to quantitative investigation of slime mold processes such as chemotactic response (Dussutour et al., 2010), biological network design (Tero et al., 2010), and foraging behaviors (Latty and Beekman, 2011).

### Implementation of the *V. officinalis* hairy root culture platform

*A. rhizogenes*-derived roots of species within the *Valerianaceae* clade have been demonstrated to stably recapitulate the biochemistry of wild type plants (Gränicher et al., 1995b; Banerjee et al., 1998; Kittipongpatana et al., 2002). Analysis of volatile constituents in the essential oil of *V. officinalis* hairy roots indicated the presence of at least 60 compounds, of which 50% (w/w) were sesquiterpene hydrocarbons and their oxygenated derivatives, as well as 3.8% monoterpene hydrocarbons and related metabolites (Gränicher et al., 1995a). The clonal nature of hairy root culture biomass addresses the genetic and chemotypic variation reported in wild type *V. officinalis* (Letchamo et al., 2004; Manzocco et al., 2011), and can be considered a precondition for the application of root-specific biological activities.

**Figure 2:**
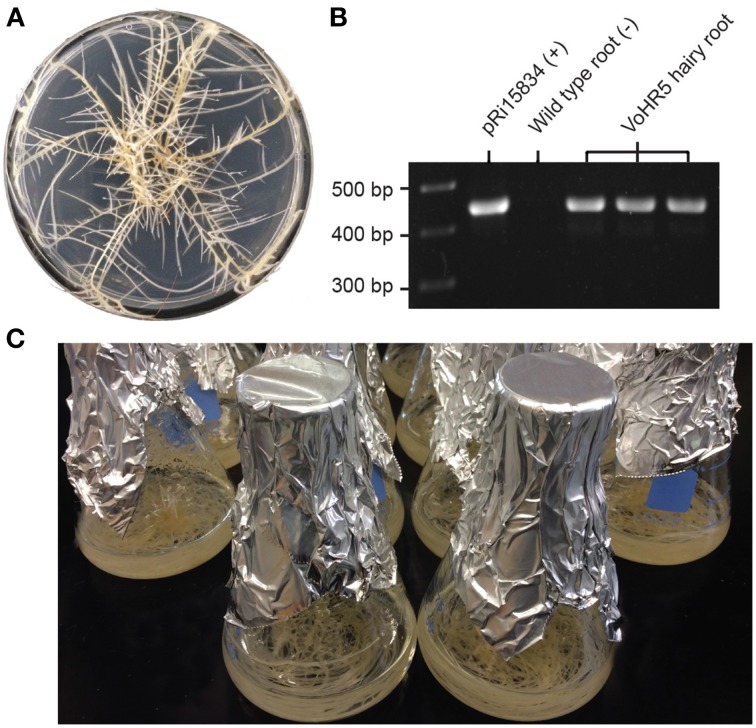
**Establishment and growth of** ***V. officinalis*** **hairy roots transformed with** ***A***. ***rhizogenes*** **harboring root-inducing plasmid pRi15384**. **(A)** Growth characteristics of the hairy root clone VoHR5 on semi-solid media. This clone featured vigorous growth and significant lateral branching that is typical of the hairy root disease phenotype. **(B)** PCR characterization of *V. officinalis* hairy root line VoHR5 for stable integration of *A. rhizogenes* root-inducing plasmid DNA. VoHR5 genomic DNA was isolated from three independent subcultures and used as template for amplification of the *rolB* gene. Plasmid pRi15834 was used as a positive control. Genomic DNA isolated from wild type plant root was used as a negative control. **(C)** Mature cultures of the VoHR5 line grown in shake-flasks before harvesting at 28 days.

To generate the transgenic roots used in this study, leaf segments of *in vitro* grown *V. officinalis* plants were used as explants for *A. rhizogenes*-mediated transformation and hairy root induction. Neoplastic roots were removed from different explants to provide independent lines and were further developed on solid media containing timentin to disinfect residual *Agrobacterium* (Figure 2A). A single root culture line, VoHR5 was selected based on fast growth and integration of *A. rhizogenes* root-inducing plasmid DNA into the plant genome. To confirm the genetic status of line VoHR5, PCR analysis was carried out on genomic template of three sequential subcultures and a 420 bp fragment of the *A. rhizogenes rolB* gene was amplified (Figure 2B). *RolB* exists on the T-DNA region of Ri plasmid pRi15834 and is diagnostic for *A. rhizogenes* DNA integration into the host plant genome (Nilsson and Olsson, 1997). The VoHR5 line was massively propagated in shake-flask culture (Figure 2C) to generate the experimental root biomass used for characterization in *P. polycephalum*.

*A. rhizogenes*-mediated genetic transformation enables manipulation of genes in addition to those responsible for the hairy root phenotype. More complex genetic engineering can be achieved by the use of *A. rhizogenes* harboring disarmed binary vectors from *Agrobacterium tumefaciens* (Hamill et al., 1987). The molecular machinery involved in the transfer and integration of *A. rhizogenes* root-inducing DNA into the plant genome can act in *trans* on T-DNA regions of binary vectors to yield recombinant hairy roots featuring genes of interest (Ono et al., 2012). This approach has been successfully applied to manipulate metabolism occurring in transformed roots of several plant species (Kuzovkina and Schneider, 2006). Plants have been engineered to influence soil microorganism signaling as exemplified by transgenic efforts to quench the quorum-sensing-dependent virulence of pathogens belonging to the genus *Pectobacterium* (Czajkowski et al., 2011). As such, recombinant *V. officinalis* hairy roots could be implemented in the future genetic engineering of tissue cultures with novel biological activities.

### *V. officinalis* hairy root culture biomass is a robust plasmodium chemoattractant

**Figure 3:**
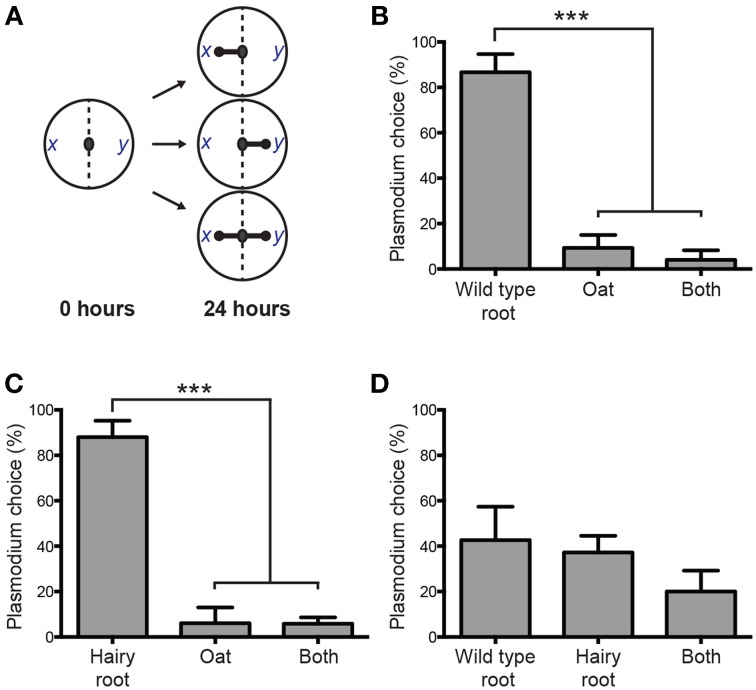
**Plasmodium binary choice chemotaxis assay of** ***V. officinalis*** **root tissues. (A)** Schematic of the experimental design for the binary choice assay. Locations of two samples are denoted by *x* and *y*. Preference for a sample is indicated by a rounded arrow and scored as a positive chemotactic event. Three outcomes are defined: propagation occurs toward sample *x*, propagation occurs toward sample *y*, or plasmodium is attracted to both *x* and *y* simultaneously. **(B)** *P. polycephalum* is strongly attracted to wild type root of the intact *V. officinalis* plant when copresented with oat, its primary nutrient source. **(C)** VoHR5 hairy root recapitulates the chemoattractive force of the wild type plant root when co-presented with oat. **(D)** Plasmodium choice between *V. officinalis* wild type plant root and *Agrobacterium*-derived hairy root was not significantly different. Data represent the means ± SD of three independent experiments containing at least twenty replicates each (^***^P≤0.001, One-Way ANOVA with Tukey's post-test with a 95% confidence interval. Detailed statistical information is provided in Table S1).

We previously used a plasmodium binary choice chemotactic assay to demonstrate the hierarchical preference for wild type *V. officinalis* root compared to other biologically active plant materials (Adamatzky, 2011). The assay quantifies plasmodium preference between two samples placed equidistant to the site of inoculation. Gradients of chemoattractants stimulate actin polymerization and subsequent protoplasmic rearrangment in foraging pseudopodia proximal to samples, directing growth toward a sample. To assess the chemoattractive potential of genetically transformed roots, experiments were undertaken to test the plasmodium's binary choice between oats, *V. officinalis* wild type plant root and *Agrobacterium*-derived hairy root (Figure 3A). *P. polycephalum* exhibited a strong chemotactic response to wild type root (Figure 3B) and hairy root (Figure 3C), recurrently propagating toward these choices when presented against oat flake, the primary nutrient source of plasmodium laboratory cultures. When presented with wild type root and hairy root simultaneously, the frequency of plasmodium choice was not significantly different (Figure 3D). These data indicate that *Agrobacterium*-derived hairy roots recapitulate the chemoattractive force of wild type plant roots and *in vitro* root cultures can be implemented as a sustainable programming source of *P. polycephalum* chemoattractants.

**Figure 4:**
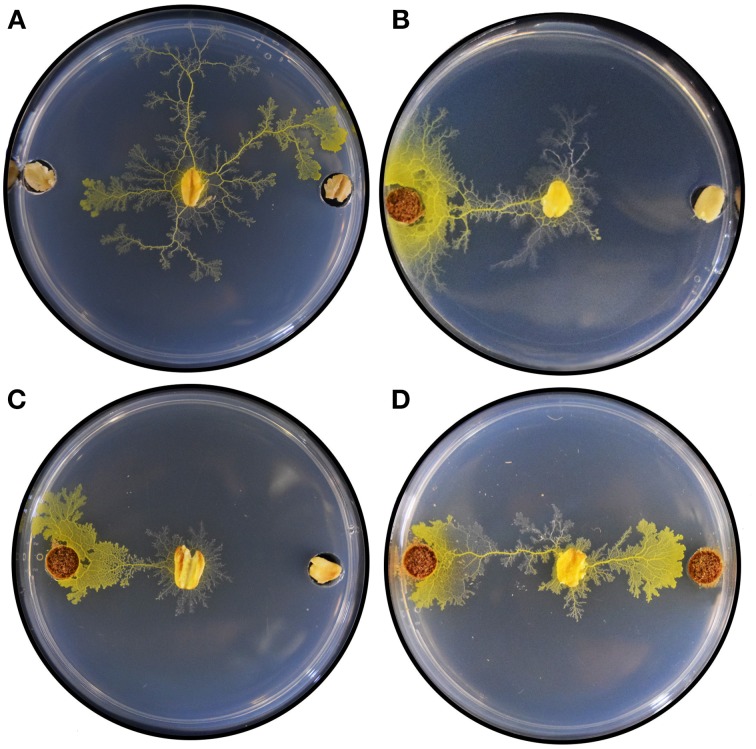
**Selected results of plasmodium binary choice experiments**. **(A)** Plasmodia inoculated onto assay plates containing oat flake in both sample locations extended concentrically onto the agar substrate, ultimately establishing a leading front toward one or both oat flakes. **(B)** Exemplary result of binary choice experiments between wild type *V. officinalis* plant root and oat. **(C)** Exemplary result of binary choice experiments between *V. officinalis* VoHR5 hairy root and oat. **(D)** Exemplary result of an equal preference event. Plasmodium chose both wild type plant root (left) and hairy root (right) simultaneously.

Figure 4 shows selected results demonstrating plasmodium behavior 24 h after initiation of the binary choice assays. As a control, we evaluated plasmodium behavior by adding a single colonized oat flake in the center of the assay plate containing oat in both sample locations. The plasmodium propagated concentrically with no apparent directionality toward either sample (Figure 4A), ultimately locating an oat flake and reorganizing its network to accommodate the nutrient source (Figure S1). When *V. officinalis* wild type plant root was co-presented with oat, plasmodia took a direct horizontal path from the inoculation site, propagating toward the root input (Figure 4B). A qualitatively similar chemotactic response was observed when VoHR5 hairy root was co-presented with oat (Figure 4C) and this root biomass induced a reliable chemotactic response featuring uniform protoplasmic arrangements in multiple independent binary choice experiments (Figure S2). Plasmodium choice was not quantitatively different when wild type and VoHR5 roots were co-presented in the same assay. Approximately 20% of these experiments featured an equal preference outcome in which two leading fronts were established and propagation occurred toward both root sample types simultaneously (Figure 4D). Such protoplasmic arrangements qualitatively support equivalent chemoattractive potential of wild type and *Agrobacterium*-derived hairy roots. While most studies on *P. polycephalum* chemotaxis employ diffusion of active compounds into a substrate shared with plasmodium, we previously found that propagation toward wild type *V. officinalis* plant root can occur along gradients of volatile attractants (Adamatzky and Costello, 2012), and confirmed that attraction to hairy root occurs via similar mechanisms (Video S1 and Figure S3).

### Hairy root biomass augments the maze-solving behavior of *P. polycephalum*

*P. polycephalum* plasmodium can compute the shortest path between selected points in a labyrinth by means of its adaptable protoplasmic network. This has been experimentally demonstrated using two methods of maze solving. In the first approach, developed by Nakagaki (2001), pieces of plasmodium are placed throughout a non-nutrient maze and a protoplasmic network spanning the whole maze is developed. Upon full colonization of the maze, the start, and end points of a path to be calculated are marked by the introduction of oat flakes into the system. Over time, tubes with higher volume of intracellular traffic become larger and tubes with lower volume are pruned back, revealing the shortest path connecting the sources of nutrients. As such, it was reported that the plasmodium computed the shortest path in a maze. However, this approach is computationally inefficient, because plasmodium inoculum must be present throughout in the system. The second approach developed by Adamatzky (2012) employs chemoattraction to navigate plasmodium toward a nutrient source located in the central chamber. Plasmodium is inoculated into the periphery of the maze and explores chamber spaces with its growing zone. Oat diffusion into the agar is detected by plasmodia sharing the same substrate and propagation is directed along the nutrient gradients toward the source. This approach is computationally efficient because it utilizes a single colonized oat flake to initiate the maze-solving task. Thus, we adopted this experimental design for the present study.

**Figure 5:**
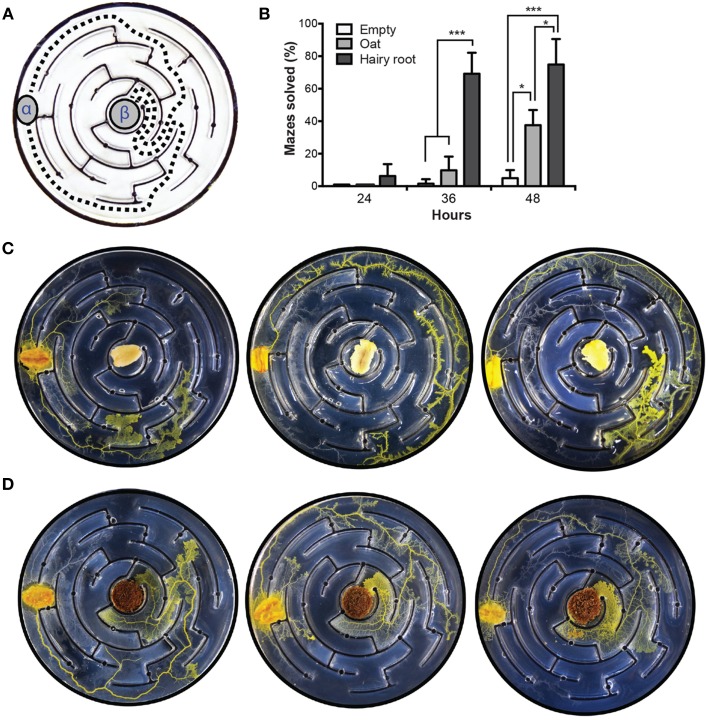
***P. polycephalum*** **maze-solving behavioral assay**. Plasmodia were inoculated into the most peripheral channel of mazes containing either oat or VoHR5 hairy root in the central chamber. Mazes with empty central chambers were used as a control and completion was scored according to the arrival at the central chamber. **(A)** Schematic representation of the two optimal shortest paths to maze completion. α, site of plasmodium inoculation, β, central chamber. **(B)** Hairy root biomass augments the maze-solving behavior of *P. polycephalum*. After 36 h, plasmodia inoculated into control and oat-containing assays completed 1.5 and 10% of the respective mazes whereas plasmodia inoculated into assays containing hairy root achieved 69% maze completion. After 48 h, plasmodia completed 5% of control and 38% of oat-containing mazes whereas 75% of hairy root mazes were completed by this time. Data represent the means ± SD of three independent experiments containing at least twenty replicates each (^*^p≤0.05, ^***^P≤0.001, One-Way ANOVA with Tukey's post-test with a 95% confidence interval. Detailed statistical information is provided in Table S2). **(C)** Exemplary results of maze-solving behavior in assay plates containing oat in the central chamber after 36 h. Plasmodia explored all possible paths with no apparent directionality. In some experiments, plasmodia established a leading front upon detection of oat diffusion into the agar but did not complete the maze at the time of sampling **(D)** Exemplary results of maze-solving behavior in assay plates containing hairy root in the central chamber after 36 h. Plasmodia were routed via the two optimal shortest paths to the central chamber end point.

To investigate the effect of hairy roots on maze-solving behavior, plasmodium was inoculated into the peripheral chamber of a labyrinth where the central chamber contained either oat or VoHR5 hairy root biomass (Figure 5A). Assays with empty central chambers were inoculated as a control. Maze completion was sampled at 24-, 36-, and 48-h post-inoculation. At the 36-h time point, the data show that maze-solving capabilities were significantly augmented by the presence of hairy root in the center chamber (Figure 5B). At 48-h, maze completion was increased by hairy root compared to empty and oat-containing central chambers, although less significantly than at 36 h. Figure 5C shows examples of plasmodium behavior when inoculated into mazes containing oat. Plasmodia established a leading front by the 36-h time point, however most completed oat-containing mazes were not solved until 48 h. Figure 5D shows examples of maze solving behavior in the presence of hairy root where plasmodium was routed via the two possible shortest paths.

These findings suggest that *V. officinalis* hairy roots may augment the plasmodium's capacity to solve shortest path problems. Such mechanisms of shortest path calculations, particularly those incorporating flexible adaptability, and re-routing, are of great interest in the development of biologically inspired path-solving algorithms (Tero et al., 2006, 2010). Self-routing and self-repairing wires are a field of application with potentially high impact on novel developments in non-silicon hardware. We previously demonstrated that protoplasmic tubes can grow long distances and propagate on electronic boards under reasonably high current and potential (Adamatzky, 2013a). Such self-routed wires can be integrated with other slime mold electronic components: oscillators (Umedachi et al., 2013), memristors (Gale et al., 2013), chemical (Whiting et al., 2014), and optical color (Adamatzky, 2013c) sensors.

### Hairy roots stimulate the oscillatory patterns of plasmodium electrical activity

**Figure 6:**
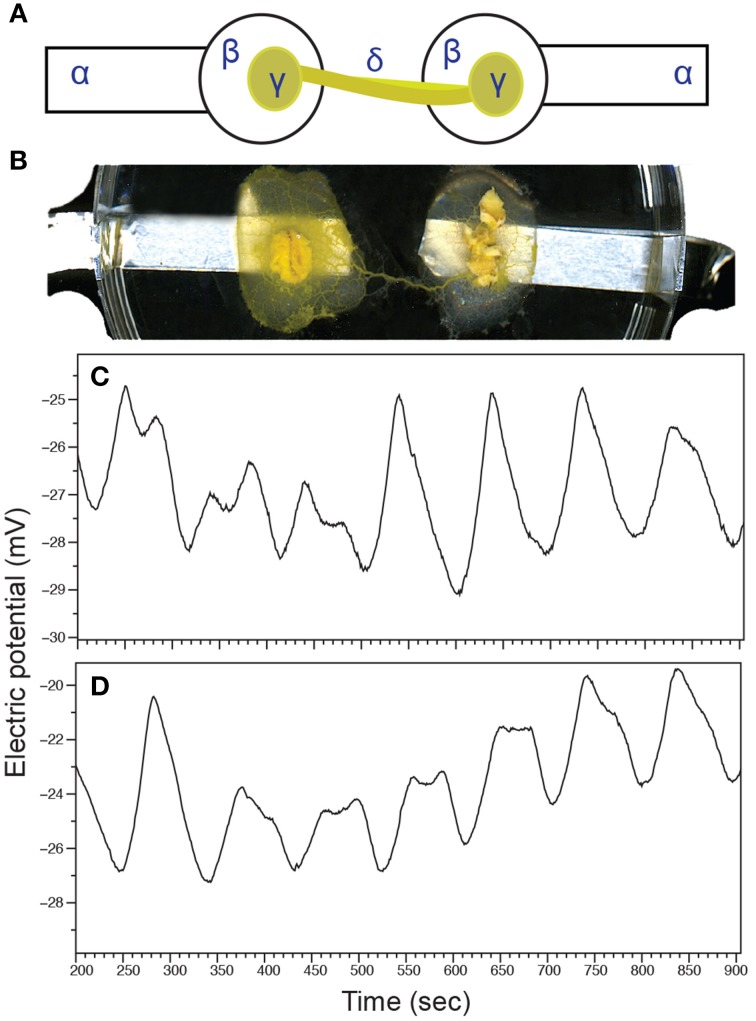
**The electrophysiological response to** ***V. officinalis*** **hairy root culture in a simple plasmodium circuit**. **(A)** Schematic representation of the experimental circuit for measuring *P. polycephalum* surface electrical activity. Plasmodium was inoculated onto agar blobs situated on aluminum electrodes. Completed circuits with a single protoplasmic tube spanning both agar blobs were used for electrical activity recording. α, aluminum electrode; β, agar blob; γ, plasmodium-colonized oat flake; δ, protoplasmic tube. **(B)** Photograph of an intact experimental circuit. A solitary protoplasmic tube connects two agar blobs situated on recording electrodes. **(C)** Exemplary patterns of electrical oscillations of undisturbed plasmodium. **(D)** Exemplary oscillation patterns of electrical activity after the introduction of VoHR5 hairy root culture biomass.

The electrophysiological response to root culture biomass was measured by recording patterns of the plasmodium's electrical activity. Undisturbed, *P. polycephalum* exhibits mostly regular patterns of oscillations of its surface electrical potential. These oscillations most likely regulate peristaltic movements of protoplasmic tubes for distribution of nutrients and spatial navigation (Heilbrunn and Daugherty, 1939; Tero et al., 2008). Calcium flux through ion channels stimulates biochemical oscillators responsible for contractile dynamics (Smith and Saldana, 1992). The slime mold's surface electrical potential oscillates with a frequency that has been correlated with shuttle streaming of cytoplasm (Smith, 1994; Yoshiyama et al., 2010). We defined alteration of basal oscillatory patterns by unique changes in frequencies of plasmodium electrical activity. Figure 6A shows a schematic of the experimental setup where a simple plasmodium circuit is completed by a single protoplasmic tube spanning two electrodes. A photograph of an intact circuit is shown in Figure 6B.

Changes in electrical activity were recorded before and after the introduction of VoHR5 hairy root into the assay containing a plasmodium circuit. Baseline electrical oscillations of control plasmodium circuits featured a dominating frequency ± SD of 0.009 ± 0.0002 Hz (Figure 6C). When hairy root was introduced into the assay, the dominating frequency of electrical oscillation changed to 0.01 ± 0.0001 Hz (Figure 6D). These findings indicate that oscillation frequencies of the plasmodium's electrical potential increase by 10% after stimulation with root culture biomass. When considering periods of oscillations these changes are transformed into values that are easily detectable by electronic devices. The dominating period of oscillations of a control plasmodium circuit was 111 s, while the dominating period of the circuit exposed to hairy root biomass is 100 s. This increase in the period of oscillations suggests stimulation by hairy root volatile constituents occurs in a manner similar to that of a biological contactless chemical sensor.

## Conclusion

Computation using *P. polycephalum* is a developing field, with most applications based on patterns governing the organism's instinctual foraging behavior and spatial awareness. However, more complex implementations may be possible by applying combinations of long- and short-range attractant and repellents. Our current findings on navigating plasmodium in a labyrinth suggest that chemoattraction to *V. officinalis* could be applied to routing the slime mold in complex, geometrically constrained, three-dimensional environments where target sites are marked by sources of root biomass. Stimulation of protoplasmic oscillations elicited by hairy roots could be used to augment the electrochemical characteristics of slime mold microfluidic devices so that unique functional elements can be realized. Preconditioning of cells in biosensor-type devices has yielded reliable and reproducible results using predetermined chemical inputs or optimized co-culture of multiple organisms (Li et al., 1994).

Other applications of plant root cultures as plasmodium modulators include future models for engineering the plant rhizosphere, an important interface where the zone of soil around the roots is influenced by root activity. Plants have evolved a multitude of strategies to manipulate soil microorganisms, lessening the impact of biotic and abiotic stresses. Exploiting these interactions might facilitate the improvement of plant fitness and productivity while minimizing input of agrochemicals into the environment (Ryan et al., 2009). Detection of plant-insect semiochemicals has been achieved using biosensors such as coupled gas chromatography-electrophysiology (GC-EAG), which exploits the olfactory sensilla situated on insect antennae. Intriguingly, the plant sesquiterpene germacrene D acts specifically on a major antennal receptor neuron of tobacco budworm moth (Røstelien et al., 2000). Contactless stimulation of *P. polycephalum* electrical activity by *V. officinalis* root suggests that plasmodium circuits could be incorporated into biosensors for evaluating agronomically significant plant-microbe interactions, such as those mediated by sesquiterpene volatiles in the rhizosphere (Akiyama et al., 2005).

### Conflict of interest statement

The authors declare that the research was conducted in the absence of any commercial or financial relationships that could be construed as a potential conflict of interest.
