# Supplementary material for: Plant hairy root cultures as plasmodium modulators of the slime mold emergent computing substrate Physarum polycephalum
Source: Front Microbiol. 2015 Jul 16;6:720. doi: 10.3389/fmicb.2015.00720 (PMC4504241; doi:10.3389/fmicb.2015.00720)
Supplement: Supplementary file 2 [file Image1.PDF]

Supplementary material

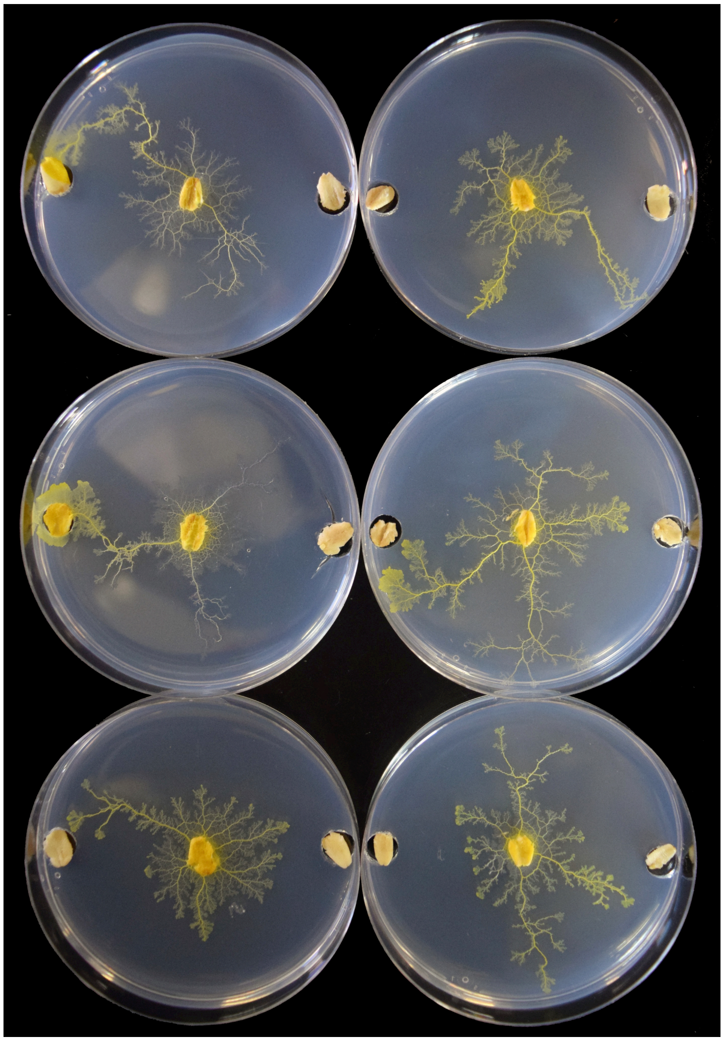

Figure S1. Typical plasmodium behavior in oat flake control binary choice chemotaxis experiments after 24 hours. Assay plates contained oat flake in both left and right sample locations. Plasmodium propagation proceeded concentrically, ultimately rearranging its network to facilitate the assimilation of the oat flakes upon detection.

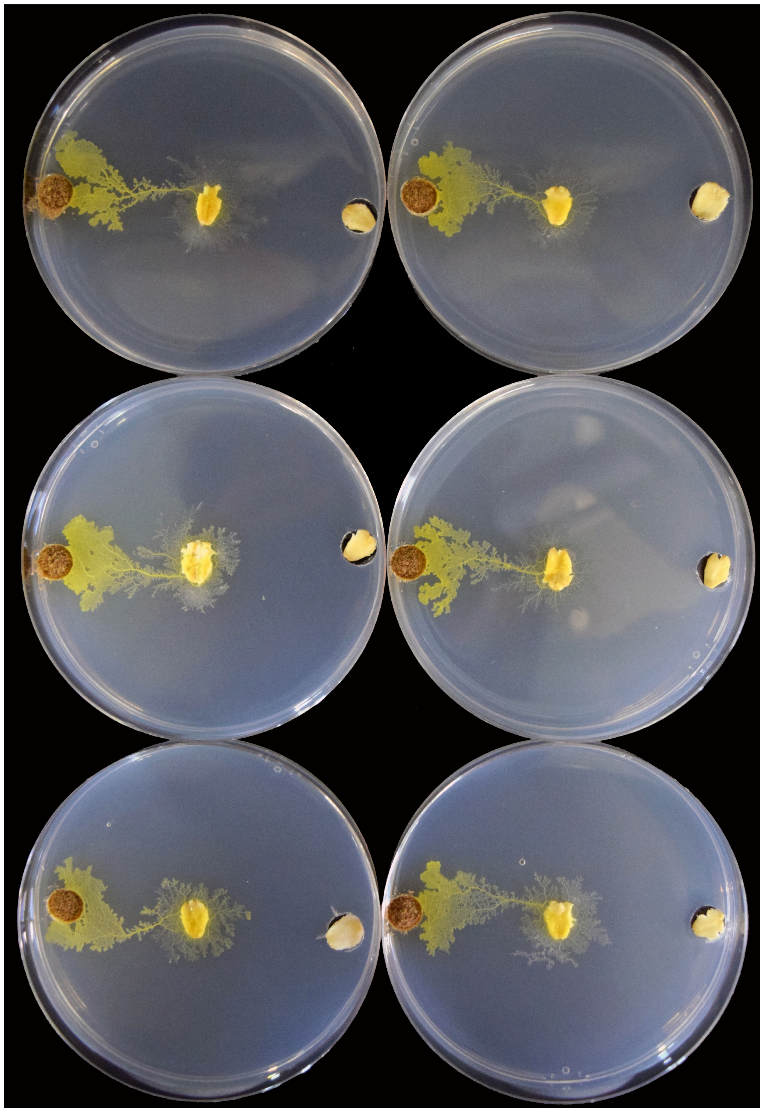

Figure S2. Typical plasmodium behavior in binary choice chemotaxis experiments between VoHR5 *V. officinalis* hairy root culture biomass (left) and oat flake (right) after 24 hours. Plasmodium recurrently propagated along a direct horizontal path toward the VoHR5 hairy root sample site, yielding a qualitatively uniform chemotactic response across multiple independent experiments.

Table S1. Plasmodium binary choice assay ANOVA analysis

Wild type root vs oat

ANOVA summary	
F	168.1
P value	< 0.0001
P value summary	****
Are differences among means statistically significant? (P < 0.05)	Yes
R square	0.9825

Tukey's multiple comparisons test	Mean Diff.	95% CI of diff.	Significant?	Summary
WT root vs. Oat	77.35	61.87 to 92.83	Yes	****
WT root vs. Both	82.59	67.11 to 98.06	Yes	****
Oat vs. Both	5.235	-10.24 to 20.71	No	ns

Test details	Mean 1	Mean 2	Mean Diff.	SE of diff.	n1	n2	q	DF
WT root vs. Oat	86.65	9.295	77.35	5.044	3	3	21.69	6
WT root vs. Both	86.65	4.06	82.59	5.044	3	3	23.15	6
Oat vs. Both	9.295	4.06	5.235	5.044	3	3	1.468	6

Hairy root vs. oat

ANOVA summary	
F	186.4
P value	< 0.0001
P value summary	****
Are differences among means statistically significant? (P < 0.05)	Yes
R square	0.9842

Tukey's multiple comparisons test	Mean Diff.	95% CI of diff.	Significant?	Summary
Hairy root vs. Oat	82	66.94 to 97.06	Yes	****
Hairy root vs. Both	82.18	67.12 to 97.25	Yes	****
Oat vs. Both	0.1818	-14.88 to 15.25	No	ns

Test details	Mean 1	Mean 2	Mean Diff.	SE of diff.	n1	n2	q	DF
Hairy root vs. Oat	88.06	6.061	82	4.909	3	3	23.62	6
Hairy root vs. Both	88.06	5.879	82.18	4.909	3	3	23.67	6
Oat vs. Both	6.061	5.879	0.1818	4.909	3	3	0.05237	6

Wild type root vs. Hairy root

ANOVA summary	
F	3.544
P value	0.0963
P value summary	ns
Are differences among means statistically significant? (P < 0.05)	No
R square	0.5416

Tukey's multiple comparisons test	Mean Diff.	95% CI of diff.	Significant?	Summary
WT root vs. Hairy root	5.435	-21.81 to 32.68	No	ns
WT root vs. Both	22.64	-4.604 to 49.89	No	ns
Hairy root vs. Both	17.21	-10.04 to 44.46	No	ns

Test details	Mean 1	Mean 2	Mean Diff.	SE of diff.	n1	n2	q	DF
WT root vs. Hairy root	42.69	37.26	5.435	8.881	3	3	0.8655	6
WT root vs. Both	42.69	20.05	22.64	8.881	3	3	3.606	6
Hairy root vs. Both	37.26	20.05	17.21	8.881	3	3	2.741	6

Table S2. Plasmodium maze solving assay ANOVA analysis

24 Hours

ANOVA summary	
F	1.922
P value	0.2265
P value summary	ns
Are differences among means statistically significant? (P < 0.05)	No
R square	0.3905

Tukey's multiple comparisons test	Mean Diff.	95% CI of diff.	Significant?	Summary
Empty vs. Oat	1	-9.557 to 11.56	No	ns
Empty vs. Hairy root	-5.277	-15.83 to 5.280	No	ns
Oat vs. Hairy root	-6.277	-16.83 to 4.280	No	ns

Test details	Mean 1	Mean 2	Mean Diff.	SE of diff.	n1	n2	q	DF
Empty vs. Oat	1	0	1	3.441	3	3	0.411	6
Empty vs. Hairy root	1	6.277	-5.277	3.441	3	3	2.169	6
Oat vs. Hairy root	0	6.277	-6.277	3.441	3	3	2.58	6

36 Hours

ANOVA summary	
F	50.19
P value	0.0002
P value summary	***
Are differences among means statistically significant? (P < 0.05)	Yes
R square	0.9436

Tukey's multiple comparisons test	Mean Diff.	95% CI of diff.	Significant?	Summary
Empty vs. Oat	-8.175	-30.79 to 14.44	No	ns
Empty vs. Hairy root	-67.66	-90.27 to -45.04	Yes	***
Oat vs. Hairy root	-59.48	-82.10 to -36.86	Yes	***

Test details	Mean 1	Mean 2	Mean Diff.	SE of diff.	n1	n2	q	DF
Empty vs. Oat	1.587	9.762	-8.175	7.371	3	3	1.568	6
Empty vs. Hairy root	1.587	69.24	-67.66	7.371	3	3	12.98	6
Oat vs. Hairy root	9.762	69.24	-59.48	7.371	3	3	11.41	6

48 hours

ANOVA summary	
F	30.75
P value	0.0007
P value summary	***
Are differences among means statistically significant? (P < 0.05)	Yes
R square	0.9111

Tukey's multiple comparisons test	Mean Diff.	95% CI of diff.	Significant?	Summary
Empty vs. Oat	-32.66	-60.04 to -5.275	Yes	*
Empty vs. Hairy root	-69.94	-97.32 to -42.55	Yes	***
Oat vs. Hairy root	-37.28	-64.66 to -9.900	Yes	*

Test details	Mean 1	Mean 2	Mean Diff.	SE of diff.	n1	n2	q	DF
Empty vs. Oat	4.921	37.58	-32.66	8.924	3	3	5.175	6
Empty vs. Hairy root	4.921	74.86	-69.94	8.924	3	3	11.08	6
Oat vs. Hairy root	37.58	74.86	-37.28	8.924	3	3	5.908	6

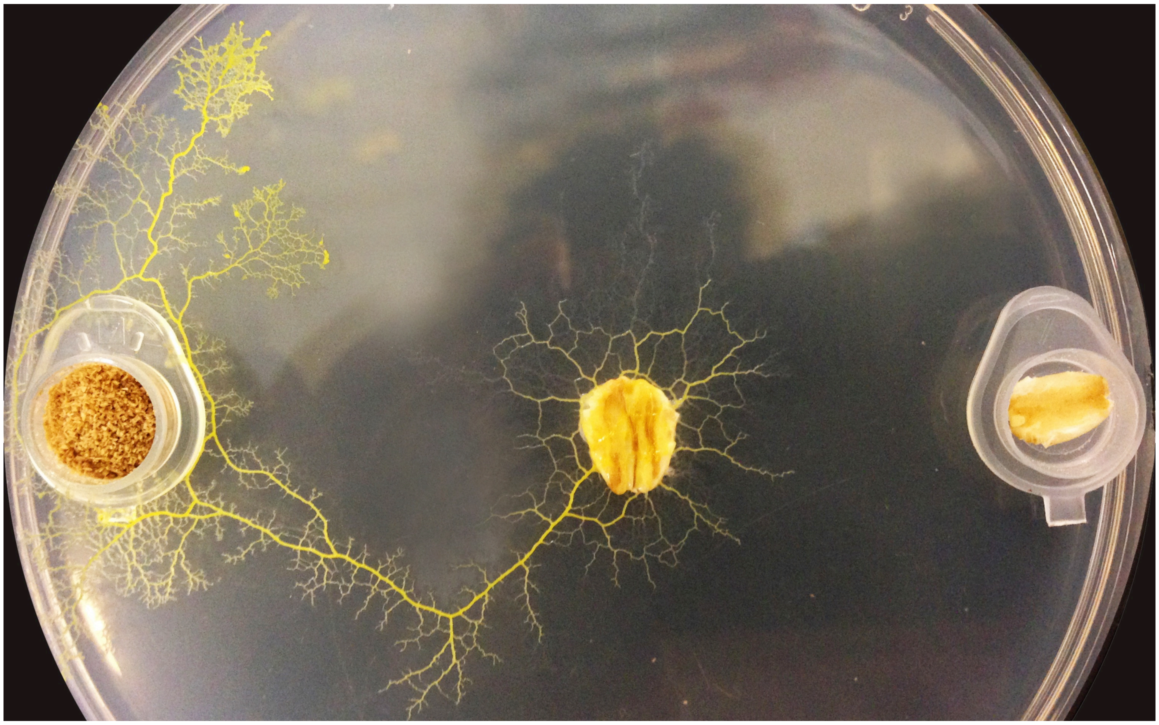

Figure S3. *P. polycephlaum* propagation toward *V. officinalis* hairy root biomass can occur via volatile chemoattractants. Photograph shows an exemplary result of a binary choice experiment between VoHR5 hairy root biomass (left) and oat flake (right) after 24 hours. Inputs were contained in plastic caps as to prevent diffusion into the agar substrate. Plasmodia repeatedly located the hairy root biomass without sharing the substrate, suggesting that *P. polycephalum* can detect volatile *V. officinalis* root compounds in a manner similar to that of a contactless chemical biosensor.
